# Structures, Electronic Properties, and Gas Permeability of 3D Pillared Silicon Carbide Nanostructures

**DOI:** 10.3390/nano12111869

**Published:** 2022-05-30

**Authors:** Onsuda Arayawut, Teerakiat Kerdcharoen, Chatchawal Wongchoosuk

**Affiliations:** 1 Department of Physics, Faculty of Science, Kasetsart University, Chatuchak, Bangkok 10900, Thailand; onsuda.ar@gmail.com; 2 Department of Physics, Faculty of Science, Mahidol University, Bangkok 10400, Thailand; teerakiat.ker@mahidol.ac.th

**Keywords:** pillared graphene, 3D SiC, SCC-DFTB, CO_2_ separation, SiC nanostructures

## Abstract

Silicon carbide (SiC) is recognized as excellent material for high power/temperature applications with a wide-band gap semiconductor. With different structures at the nanosize scale, SiC nanomaterials offer outstanding mechanical, physical, and chemical properties leading to a variety of applications. In this work, new 3D pillared SiC nanostructures have been designed and investigated based on self-consistent charge density functional tight-binding (SCC-DFTB) including Van der Waals dispersion corrections. The structural and electronic properties of 3D pillared SiC nanostructures with effects of diameters and pillar lengths have been studied and compared with 3D pillared graphene nanostructures. The permeability of small gas molecules including H_2_O, CO_2_, N_2_, NO, O_2_, and NO_2_ have been demonstrated with different orientations into the 3D pillared SiC nanostructures. The promising candidate of 3D pillared SiC nanostructures for gas molecule separation application at room temperature is highlighted.

## 1. Introduction

Silicon carbide (SiC) is recognized as an excellent material for high power/temperature applications with a wide-band gap semiconductor ranging from 2.0 to 7.0 eV. SiC has a high Young’s modulus (400–500 GPa), good thermal conductivity (5 W cm^−1^ K^−1^), large electron mobility (800 cm^2^ V^−1^ s^−1^), high electric field breakdown strength (>1 MV cm^−1^), high chemical stability, and is biocompatible [1,2]. The reduction in SiC size to the nanoscale level provides outstanding mechanical, physical, and chemical properties due to quantum confinement effects. For example, Chen et al. [3] synthesized 1.9–4.5 nm SiC quantum dots (QDs) via an electrochemical etching method and used them as emitting layers in LEDs. They exhibited a significant enhancement in electroluminescence performances, dominated by carrier transports from quantum tunneling at a low voltage. Cao et al. [4] prepared SiC QDs (average lateral particle size of 3.0 nm) by using a hydrothermal method. Their interesting biological applications of the SiC QDs were demonstrated through the detection of NHDF, HeLa, and A549 cells. Yildirim et al. [5] grew SiC nanowires (diameters of 30–90 nm with lengths up to 50 µm) by using RF-inducted metal-organic chemical vapor deposition and fabricated a self-powered SiC nanowire-based photodetector. The SiC nanowire-based device exhibited excellent detectivity of 7.2 × 10^10^ cm Hz^1/2^ W^−1^ and high external quantum efficiency of 83% without any external power. Zekentes et al. [6] reported a comprehensive review of SiC nanowire-based field-effect-transistors showing superior properties for integrated circuits and sensing applications. Sun et al. [7] presented a synthesis of 2D SiC nanosheets via the carbothermal reduction process and its application for humidity sensing. The SiC nanosheet-based humidity sensor showed high selectivity and sensitivity to a wide range of relative humidity (11–95%) with fast response and recovery times (3 s). The current research has displayed the successful synthesis and application of SiC nanomaterials ranging from 0D (QDs) to 1D (nanowires) and 2D (nanosheet). However, up to now, theoretical and experimental studies on 3D pillared SiC nanostructures are still limited. 

In recent years, air pollution has become a serious public health concern in many countries around the world. Reduction and utilization of emitted gases/volatile organic compounds (VOCs) have had an increasing interest to prevent and solve the worst impacts on the natural environment, including global warming, climate change, and human health effects [8,9,10,11,12,13,14,15,16,17,18,19,20]. One of the important keys to high efficient gas reduction and utilization is to capture or separate a target gas molecule from gas/VOCs mixtures such as separation of N_2_ in ammonia plants [21,22], CO_2_ separation in coal-fired power plants [23,24], removal of VOCs from O_2_/N_2_ streams [25], or separation of toxic chemicals from H_2_O [26,27]. Several organic molecular sieve membranes have been researched and investigated for chemical separations in many applications [28]. One of the interesting materials is SiC, which can be considered a potential membrane for gas separation because it is able to operate under harsh environments such as high pressure and high temperatures with aggressive chemicals [29,30].

In this work, we model and present new 3D pillared SiC nanostructures based on self-consistent charge density functional tight-binding (SCC-DFTB) including Van der Waals dispersion corrections. The structural and electronic properties of the 3D pillared SiC nanostructures have been investigated and compared with 3D pillared graphene (pure C) nanostructures. The permeability of small gas molecules including H_2_O, CO_2_, N_2_, NO, O_2_, and NO_2_ have been demonstrated with different orientations into the 3D pillared SiC nanostructures as well as the effects of diameters. To our best knowledge, this is the first work to investigate the structure, electronic properties, and gas permeability of the 3D pillared SiC nanostructures based on the SCC-DFTB method. The results show that the 3D pillared SiC nanostructures are promising candidates for gas molecule separation application at room temperature. 

## 2. Simulation Details

### 2.1. SCC-DFTB Method

The SCC-DFTB method is based on a second-order expansion of the Kohn–Sham total energy in density functional theory (DFT) around a reference density ($\rho_{0}$) with respect to charge density fluctuations (Δρ) [31,32,33]. The total energy ($E_{SCC-DFTB}$) can be written by the following equation:

(1) $$E_{SCC-DFTB}=\underset{i\mu v}{\sum }c_{\mu }^{i}c_{v}^{i}H_{\mu v}^{0}\left[\rho_{0}\right]+\underset{A>B}{\sum }E_{AB}^{rep}\left[\rho_{0}\right]+\frac{1}{2}\underset{AB}{\sum }\gamma_{AB}\Delta q_{A}\Delta q_{B}$$

where *A* and *B* represent atoms, μ and ν are atomic orbitals, $H_{\mu \nu }^{0}$ is the Kohn–Sham Hamiltonian, $c_{\mu }^{i}$ and $c_{\nu }^{i}$ denote the molecular orbital coefficients, $E_{AB}^{rep}$ is the two-body repulsive potential, $\gamma_{AB}$ describes a distance-dependent function for charge interactions, and $\Delta q_{A}$ and $\Delta q_{B}$ denote the induced charges.

It should be noted that SCC-DFTB can predict structures, electronic properties, and energies in excellent agreement with DFT methods as well as faster calculation time more than ~100–1000 times [34,35,36]. Moreover, the results obtained by SCC-DFTB were consistent with experimental observations [37,38,39]. SCC-DFTB including weak interactions such as Van der Waals dispersion and H-bonding with pbc-0-3 parameters [40,41] has been thus selected for this study. 

### 2.2. Models of 3D Pillared SiC Nanostructures and Calculation Details

**Figure 1:**
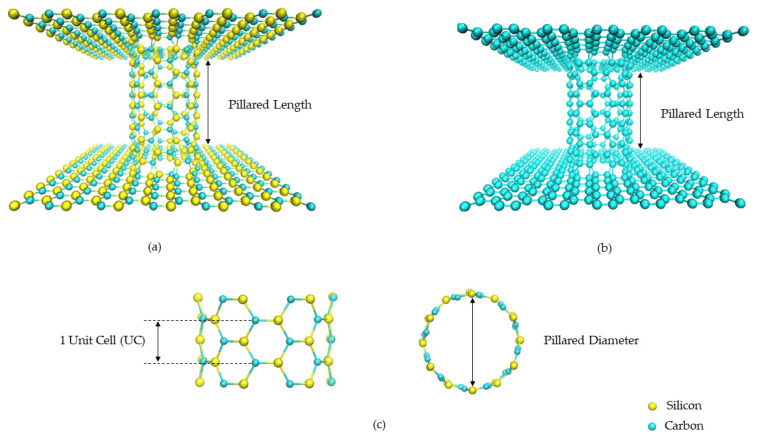
Models of 3D pillared (**a**) SiC and (**b**) C nanostructures. (**c**) Definition of pillar length in one unit cell and pillar diameter.

The 3D pillared SiC nanostructures were modeled and created by using an armchair SiC single-walled nanotube sandwiched with two zigzag SiC nanosheets, as shown in Figure 1a. The 3D pillared C nanostructures were also built under similar conditions in order to compare their structural and electronic properties with the 3D pillared SiC nanostructures, as shown in Figure 1b. It should be noted that the edges of the nanostructures were not saturated by H atoms because they affect the structural and electronic properties. The initial Si-C and C-C bond lengths were set at 1.786 Å [42] and 1.421 Å [43] for 3D pillared SiC and C nanostructures, respectively. The initial size of the SiC nanosheet is *L*_x_ = 3.605 nm and *L*_y_ = 3.076 nm whereas the pristine C nanosheets are *L*_x_ = 2.849 nm and *L*_y_ = 2.432 nm. Because of different bond lengths, the pillar length is defined as a unit cell. One unit cell of 3D pillared SiC and C nanostructures and their length before geometry optimization are displayed in Figure 1c. To create the joints connecting nanosheets and nanotubes, Euler’s rule is used to verify the validity of the polygons at the junction [44,45] according to the equation:

*F* + *V* = *E* + 2 − 2*G* (2)

where *F*, *V*, *E*, and *G* are the number of faces, vertices, edges, and genus, respectively.

The visual molecular dynamics (VMD) program was employed for the visualization of molecules [46]. The diameters of armchair nanotubes were also varied, including (3,3), (4,4), (5,5), and (6,6). All models were fully optimized without any constraints until the atomic forces were less than 1.0 × 10^−4^ Hartree/Bohr. To improve SCC convergence and add the thermal electronic excitation effect, the electron temperature was set at 300 K [47]. The SCC tolerance was set at 10^–6^ au. It means that the convergence of calculation is reached if the energy difference is less than 2.72 × 10^−5^ eV. All SCC-DFTB calculations were performed using the DFTB+ [48]. Command codes for running full optimization and gas interaction are displayed in Appendices SA and SB in the Supporting Information. The stability of nanostructures can be evaluated via the average binding energy ($E_{avg}$) by using the following equations [49]:

(3) $$E_{avg}(3D-SiC)=\frac{N_{C}E(C)+N_{Si}E(Si)-E_{tot}(3D-SiC)}{N_{C}+N_{Si}}\;\mathrm{for}\;3D\text{-}\mathrm{SiC}\;\mathrm{models}$$

(4) $$E_{avg}(3D-C)=\frac{N_{C}E(C)-E_{tot}(3D-C)}{N_{C}}\;\mathrm{for}\;3D\text{-}C\;\mathrm{models}\;$$

where *N_C_* is the total carbon atomic number, *N_Si_* is the total silicon atomic numbers, *E*(*C*) and *E*(*Si*) are the energy of one carbon and one silicon atom, respectively, *E_tot_*(3*D-SiC*) is the total energy of 3D pillared SiC nanostructures, and *E_tot_*(3*D-C*) represents the total energy of 3D pillared *C* nanostructures. 

The energy gap (*E*_gap_) can be estimated from the difference between the highest occupied molecular orbital (HOMO) and the lowest unoccupied molecular orbital (LUMO) according to the equation:

*E*_gap_ = LUMO − HOMO (5)

### 2.3. Interaction of 3D Pillared SiC Nanostructure and Gas Molecules

**Figure 2:**
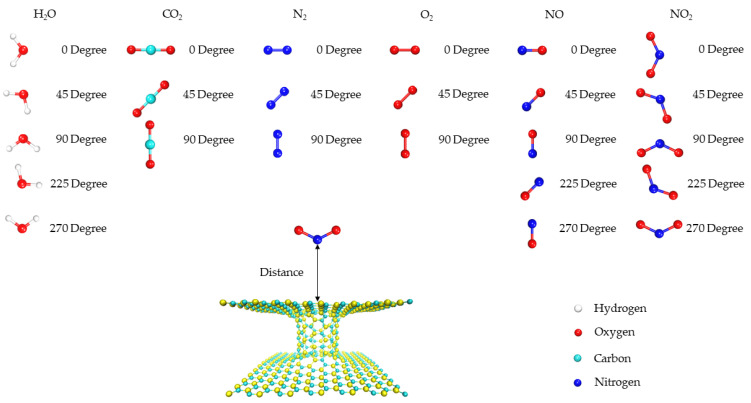
Models for the diffusion of gas molecules with various orientations into the 3D pillared SiC nanostructure.

To demonstrate an application of the 3D pillared SiC nanostructure, small gas molecules including H_2_O, CO_2_, N_2_, NO, O_2_, and NO_2_ were placed on the top of the 3D pillared SiC nanostructure around the center of the pillar as shown in Figure 2. The distance (D) between gas molecules and 3D pillared SiC nanostructure can be defined as the starting point (0 Å) around the entrance channel. The negative and positive distances represent the inside and outside of the pillared SiC nanostructure, respectively. The gas molecules with various orientations move into the 3D pillared SiC nanostructures with different diameters. The interaction energy ($E_{int}$) can be calculated via the equation:

(6) $$E_{int}=E_{tot}(3DSiC+gas_{}molecule)-E_{tot}(3DSiC)-E_{tot}(gas_{}molecule)$$

where *E_tot_*(3*DSiC* + *gas molecule*) is the total energy of 3D pillared SiC nanostructure with a gas molecule, *E_tot_*(3*DSiC*) is the total energy of individual 3D pillared SiC nanostructure, and *E_tot_*(*gas molecule*) is the total energy of an individual gas molecule.

## 3. Results and Discussion

### 3.1. Structural Properties of 3D Pillared SiC Nanostructures

**Figure 3:**
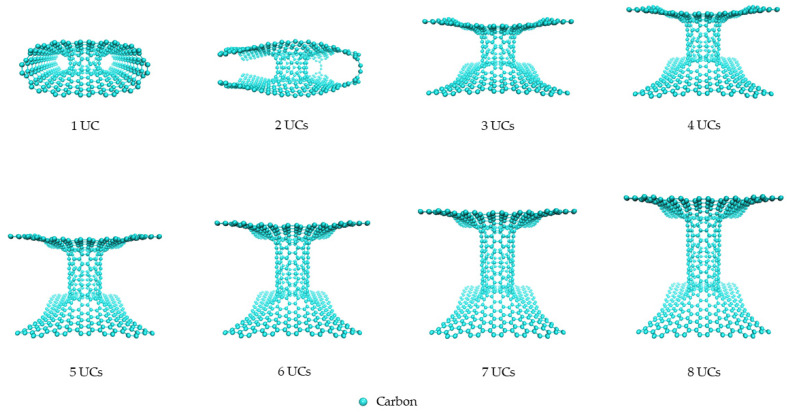
The 3D pillared C (6,6) nanostructures with various pillar lengths after full geometry optimization by the SCC-DFTB method.

**Figure 4:**
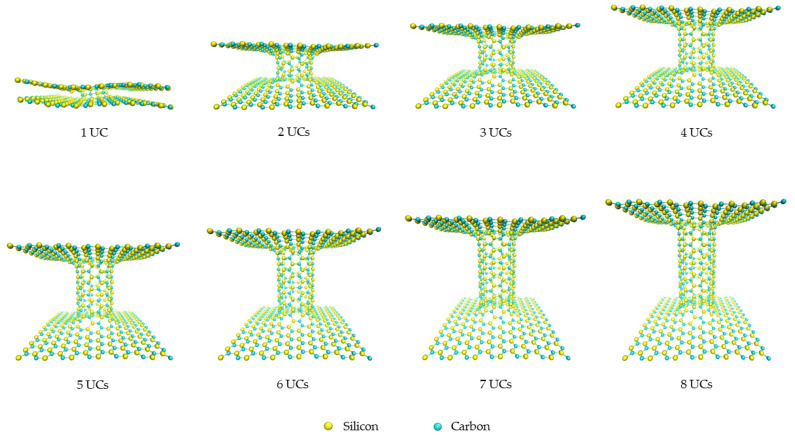
The 3D pillared SiC (6,6) nanostructures with various pillar lengths after full geometry optimization by the SCC-DFTB method.

The 3D pillared graphene (pure C) and SiC nanostructures with various pillar lengths after full geometry optimization by the SCC-DFTB method are displayed in Figure 3 and Figure 4, respectively. Interesting, graphene sheets of the 3D pillared graphene nanostructures with 1 and 2 UCs exhibit a bending of sheet edges leading to perfect roll up for the 1 UC model as shown in Figure 3. A roll-up of graphene sheet edges comes from a short initial pillar length (2.46 Å) before SCC-DFTB optimization that allows the π-orbitals to form predominantly contributions from the Van der Waals attractive forces [50,51]. With increasing the pillar length to 2 UCs, the bending of graphene sheets still occurs. However, the attractive forces of graphene sheet edges can be less affected when the initial pillar length increases more than 3 UCs (7.64 Å) and the pillar length of three UCs is extended to 13.06 Å after SCC-DFTB optimization. The structures of 3D pillared graphene seem to be stable and no defect occurs when the pillar length is granter than 3 UCs. In the case of the 3D pillared SiC nanostructures, no roll-up of SiC nanosheet exists in all short pillar lengths, as shown in Figure 4. The SiC nanosheets were found to have high in-plane stiffness, energetical stability, and a 100% planar structure that contributes to synergistic effects between sp^2^ hybridization of C atoms in its planar form and sp^3^ hybrid in Si atoms [52]. A full list of pillar diameters and pillar lengths of the 3D pillared C and SiC nanostructures before and after geometry optimization by the SCC-DFTB method is summarized in Table 1 and Table 2. 

**Figure 5:**
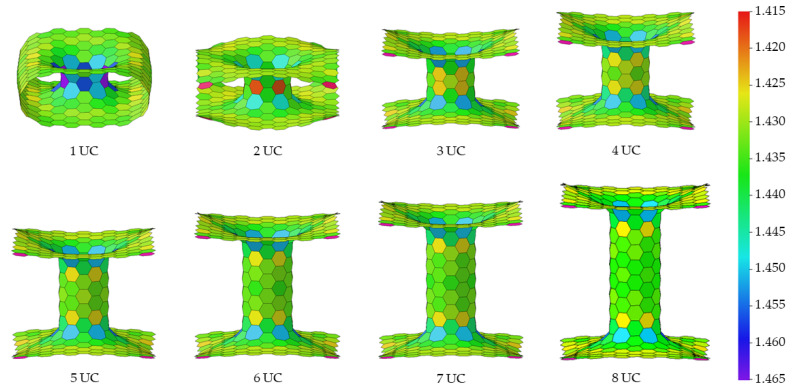
The MBL analysis of the 3D pillared C (6,6) nanostructures with various pillar lengths after full geometry optimization.

**Figure 6:**
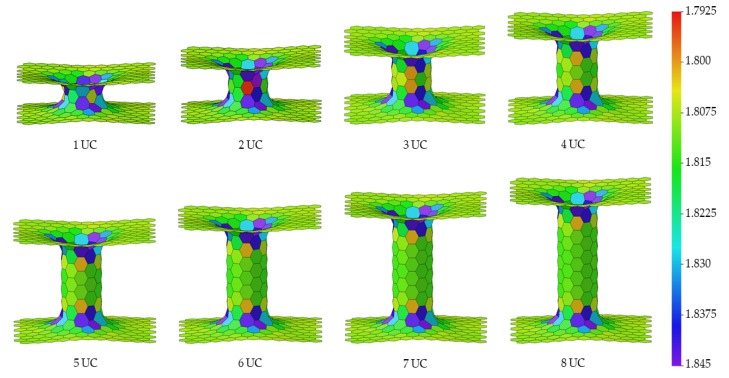
The MBL analysis of the 3D pillared SiC (6,6) nanostructures with various pillar lengths after full geometry optimization.

To analyze more details of the 3D pillared C and SiC nanostructures, the ring anisotropy and size of the 3D pillared C and SiC nanostructures were investigated via mean bond length (*MBL*) analysis [53,54], as displayed in Figure 5 and Figure 6, respectively. The *MBL* can be defined as the mean value for all bonds composing a particular ring according to the equation:

(7) $$MBL=\frac{1}{N}\sum_{i=1}^{N}b_{i}$$

where $b_{i}$ is each bond in the ring. *N* is the number of bonds in the ring.

**Figure 7:**
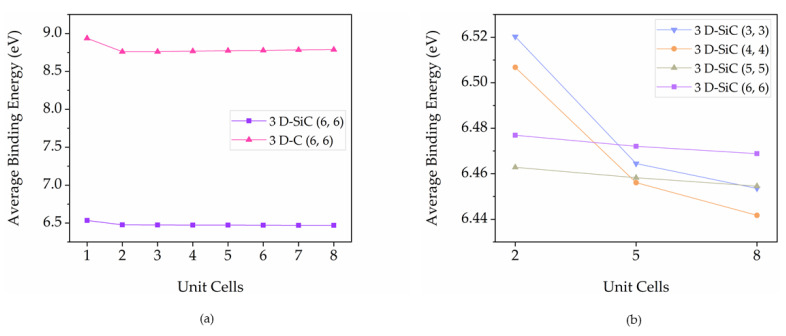
The average binding energies of (**a**) 3D pillared C and SiC nanostructures as a function of pillar length and (**b**) 3D pillared SiC nanostructures as a function of pillar diameter.

The mean bond lengths of 3D pillared C and SiC nanostructures are varied with the location of nanostructures. They extend from the original bonding after geometry optimization by the SCC-DFTB method. At interconnections between sheets and tubes, the mean bond lengths exhibit high values due to strain relaxation. All sheets show high homogeneous mean bond length while mean bond lengths of tubes exhibit high uniform with increasing pillar length. At the different pillar diameters, the mean bond length of SiC nanostructure also displays high uniform without any holes and defects, as shown in Figure S1 in the Supporting Information. The average binding energies of 3D pillared C and SiC nanostructures with various pillar lengths are shown in Figure 7a. The 3D pillared C nanostructures clearly exhibit higher average binding energies than the SiC nanostructures indicating the high structural stability of 3D pillared C nanostructures over 3D SiC nanostructures. This result refers to a difficulty in the experimental synthesis of 3D SiC nanostructures, which is why no research on the perfect growth of 3D SiC nanostructures is available compared with 3D pillared C nanostructures [55]. However, based on these average binding energies of 3D pillared SiC nanostructures, it has a high enough structural stability to provide a possibility for the experimental growth of 3D SiC nanostructures. To study the effect of pillar diameter on stability, the average binding energies of 3D SiC nanostructures with different pillar diameters are shown in Figure 7b. For short lengths (2 UCs, 12.00 Å), the average binding energies of different pillar diameters are 6.46–6.52 eV. In the case of long lengths (8 UCs, 31.34 Å), the average binding energies of different pillar diameters are 6.44–6.47 eV. They exhibit a very small difference in average binding energies (<0.1 eV for all models). It suggests that these finite pillar diameters and lengths are less affected by the formation of 3D SiC nanostructures. 

### 3.2. Electronic Properties of 3D Pillared SiC Nanostructures

**Figure 8:**
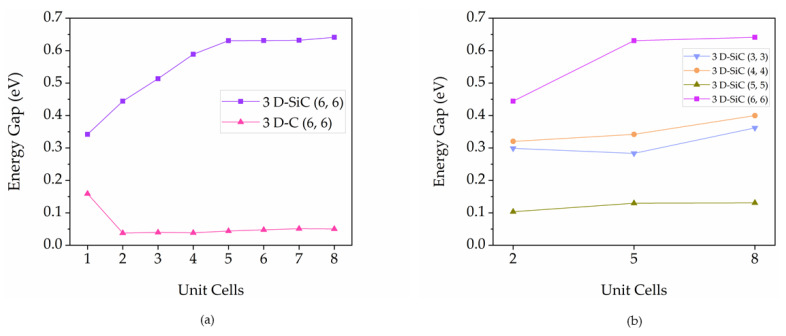
HOMO-LUMO energy gaps of (**a**) 3D pillared C and SiC nanostructures as a function of pillar length and (**b**) 3D pillared SiC nanostructures as a function of pillar diameter.

The calculated energy gaps of 3D pillared C and SiC nanostructures as a function of the pillar lengths are displayed in Figure 8a. The 3D pillared C nanostructures have nearly zero energy gaps, suggesting metallic behavior. In the case of 3D pillared SiC nanostructures, the energy gaps tend to converge into a value when pillar lengths increase. In fact, Si-C bonds in 3D-SiC nanostructures contain ionic-type bonding only that localizes the electronic states compared to the covalent C-C bonds in 3D pillared C nanostructures leading to the semiconducting behavior of 3D pillared SiC nanostructures. To investigate the effects of pillar diameter, the energy gaps of 3D pillared SiC nanostructures with different pillar diameters are shown in Figure 8b. In order to exclude 3D pillared SiC nanostructures with (5,5) diameter, the energy gaps increase as pillar diameters increase. When the pillar diameters increase, the evolution of curvature-induced π−σ hybridization decreases, resulting in higher energy gaps. It should be noted that the band gap of SiC nanostructures behaves as a non-monotonic function of their diameter [56]. The 3D pillared SiC nanostructures with (5,5) diameter did not show any trend of energy gaps. The pillar diameter strongly affects the electronic properties of the 3D pillared SiC nanostructure. These results are in good agreement with the diameter effects of SiC nanotubes on electronic properties [57,58].

**Figure 9:**
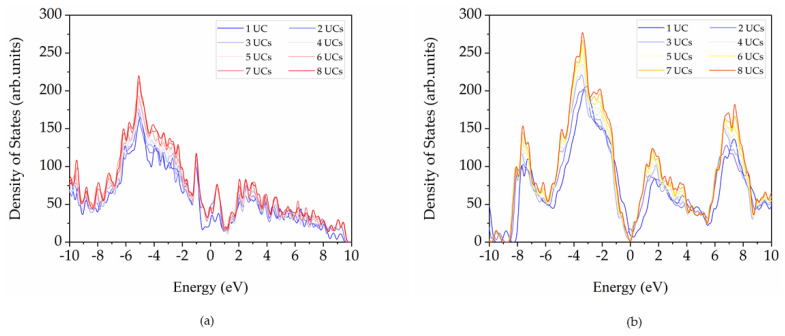
Density of states for 3D pillared (**a**) C and (**b**) SiC nanostructures as a function of the pillar lengths (Fermi level = 0 eV).

Total densities of states (DOSs) of 3D pillared C and SiC nanostructures as a function of the pillar lengths were calculated as illustrated in Figure 9a,b, respectively. The 3D pillared C nanostructures exhibit the finite density of states at the Fermi level confirming the conducting-type material. The 3D pillared SiC nanostructures show semiconducting DOSs, with the sharpest of the DOSs located near the edge of the valence bands. It is dominated by the hybridization of C (p) and Si (p) states. With increasing pillar lengths, DOSs increase due to an increase of total atoms with no significant altering of densities of states. Only 1 UC, the DOS curves are slightly shifted to lower energies that indicate more stability according to the results from average binding energies.

### 3.3. Gas Permeability of 3D Pillared SiC Nanostructures

The diffusions of several small gas molecules such as H_2_O, CO_2_, N_2_, NO, O_2_, and NO_2_ into the 3D pillared SiC nanostructures were investigated with various pillar diameters: (3,3), (4,4), (5,5), and (6,6). The pillar length of three unit cells was selected because this length was enough to represent the SiC–gas interaction behaviors as shown in Figure S2 in the Supporting Information. Similar behaviors were found for all pillar lengths of 3D pillared SiC nanostructures referring to the negligible pillar length effects on gas interactions. It should be noted that the channel of 3D pillared SiC nanostructures exhibits high symmetry after geometry optimization by the SCC-DFTB. Thus, the diffusion trajectories of all molecule gases were set at the center of pillar diameter and moved from outside into the center of mass of the 3D pillared SiC nanostructures only (half of the pillar length). The diameters of the gas molecules after geometry optimization by the SCC-DFTB method are summarized in Table 3. 

For the smallest pillar diameter (3,3) (6.25 Å), no significant SiC–gas interactions for all configurations at distances of more than 4 Å were found, as shown in Figure S3 in the Supporting Information. Although the diameters of all gas molecules (1.12–2.34 Å) are much less than the smallest pillar diameter (3,3) of 3D pillared SiC nanostructure, the repulsive force clearly increases in all configurations at a distance less than 2 Å. The interaction energies around the entrance channel (0 Å) of the 3D pillared SiC nanostructures are in the range of 6.9–46.7 eV. These results indicate that gas molecules cannot enter the 3D pillared SiC nanostructures with (3,3) pillar diameters.

In the case of pillar diameter (4,4) (8.80 Å), the potential surface energies are still highly similar to the smallest pillar diameter (3,3), as shown in Figure S4 in the Supporting Information. Compared with the (3,3) pillar diameter, the potential surface barriers seem to be decreased. At the entrance channel (0 Å), the highest interaction energy found from CO_2_ gas interacting with the (4,4) pillar diameter is 2.9 eV, while the interaction energy of the (3,3) pillar diameter to CO_2_ is high as 46.7 eV. At the inside of the pillared SiC nanostructure (−2 to −3 Å), the interaction energies of pillar diameter (4,4) are in the range of 5.6–35.0 eV reduced from the (3,3) pillar diameter’s maximum of 246.6 eV. At similar to pillar diameter (3,3), all gas molecules cannot diffuse into the 3D pillared SiC nanostructures with pillar diameter (4,4), since the large repulsive interactions dominate the potential energy surface.

**Figure 10:**
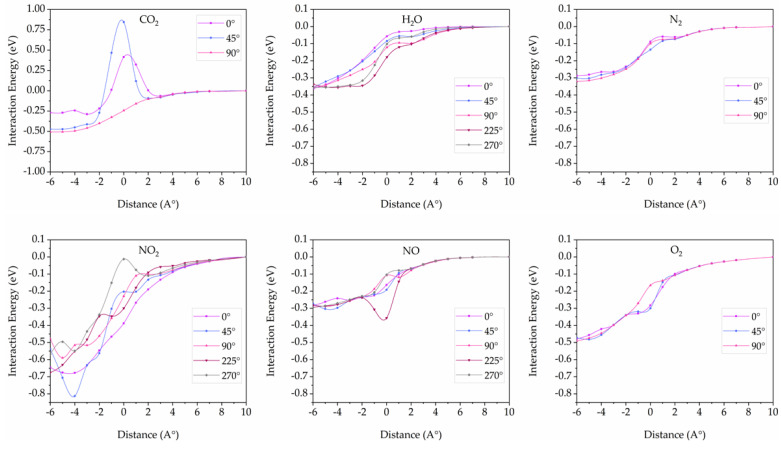
Interaction energies between 3D pillared SiC (5,5) nanostructures and gas molecules with various orientations as a function of distance.

**Figure 11:**
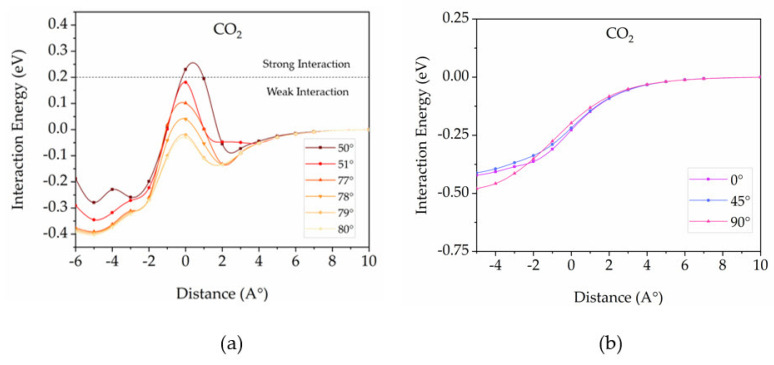
Interaction energies between (**a**) 3D pillared SiC (5,5) nanostructure and CO_2_ gas molecules with some fine orientations, (**b**) 3D pillared C (6,6) nanostructure and CO_2_ gas molecule with various orientations as a function of distance.

For the (5,5) pillar diameter (10.59 Å), the potential surface energies decrease with all gas configurations except CO_2_, as displayed in Figure 10. It suggests that small gas molecules such as H_2_O, N_2_, NO, O_2_, and NO_2_ can intercalate into the 3D pillared SiC nanostructures with pillar diameter (5,5) because attractive interactions become the most contribution. In the case of CO_2_, one configuration (90 degrees) can diffuse into the 3D pillared SiC nanostructures. It is not a surprising result because the 90-degree configuration of CO_2_ aligns as a straight line into the pillared direction leading to the smallest mean free path. The repulsive interactions at the 0- and 45-degree configuration still occur around the entrance channel (0 Å) of the 3D pillared SiC nanostructures that can help to hinder CO_2_ entrance into the interior of 3D pillared SiC nanostructures. The formation of the potential energy barrier may be attributed to the natural potential energy surface and higher activation energy of CO_2_ [59,60,61]. To find the exact angle for possible CO_2_ intercalation into the 3D pillared SiC (5,5) nanostructure, additional calculations were done as shown in Figure 11a. At the entrance channel, interaction energies exhibit positive energies at the 77- and 78-degree configurations and negative values at the 79- and 80-degree configurations. However, positive energies at the 77- and 78-degree configurations are quite small. The potential energy surface may not be enough to hinder the CO_2_ intercalation into the 3D pillared SiC nanostructure. Therefore, the cut-off energy for determining the diffusion into the 3D pillared SiC nanostructures can be defined as 0.2 eV in this work. It should be noted that the 0.2 eV was used as a reference interaction energy for the classification of physisorption/chemisorption [62,63,64,65]. Physisorption is a weak interaction while chemisorption involves a chemical reaction between the surface and the adsorbate with much stronger bonding bonds such as covalent bonding, strong electrostatic, and ionic bonding (>0.2 eV). Based on the cut-off interaction energy of 0.2 eV, they suggest that the starting angle for CO_2_ intercalation into the 3D pillared SiC (5,5) nanostructure is the 51-degree configuration. 

To clarify the performance of the 3D pillared SiC (5,5) nanostructure for CO_2_ separation application, the interaction energies between 3D pillared C (6,6) nanostructure having a diameter of 9.92 Å and CO_2_ gas molecules with various orientations were investigated as shown in Figure 11b. It should be noted that the diameters of optimized 3D pillared pure C and SiC nanostructures cannot be treated equally due to the different natures C-C and Si-C bonding. The 3D pillared C (6,6) nanostructure was selected to compare the interaction energy results with the 3D pillared SiC (5,5) nanostructure because the diameter of the optimized C (6,6) nanostructure (9.92 Å) is close to the diameter of optimized SiC (5,5) nanostructure (10.59 Å). In addition, the effective diameter of the nanostructures affected by the radius of the atoms forming the channel may be similar since the Van der Waals radius of Si and C atoms were ~2.1 Å and C ~1.7 Å, respectively. As shown in Figure 11b, there are not any potential surface berries in all configurations, indicating that the separation of CO_2_ does not occur for the 3D pillared C (6,6) nanostructure. The pillar diameter size not only plays an important parameter for gas separation but also the type of material. The different elements in nanomaterials lead to different charges, orbital, dipole moment, force field, etc., that make them have unique interactions with gas molecules. Therefore, the pillared SiC nanostructure with the pillar diameter (5,5) is able to use as a CO_2_ separation application from small gas molecules with an efficiency of 56%. The efficiency is defined as the number of CO_2_ degree configurations that cannot enter into the 3D pillared SiC nanostructures based on cut-off interaction energy of >0.2 eV (numbers of 51 from 0 to 50 degrees) per the number of all degree configurations (numbers of 91 from 0 to 90 degrees). Moreover, the pillared SiC nanostructure with the (5,5) pillar diameter can be applied for a smart nanomaterial that filters the small gas molecules including H_2_O, N_2_, NO, O_2_, and NO_2_ from many bigger volatile organic compounds (VOCs)/gases for devices/processes/applications. To confirm this application, the interaction energies between the 3D pillared SiC (5,5) nanostructure and VOCs such as acetone, toluene, and xylene were investigated and displayed in Figure S5 in the Supporting Information. The results clearly show high potential surface berries for all VOCs with parallel configuration suggesting a potential to separate VOCs/high molecular weight gas molecules for the 3D pillared SiC (5,5) nanostructure with an efficiency of 100% (no VOCs can diffuse into 3D pillared SiC (5,5) nanostructure).

**Figure 12:**
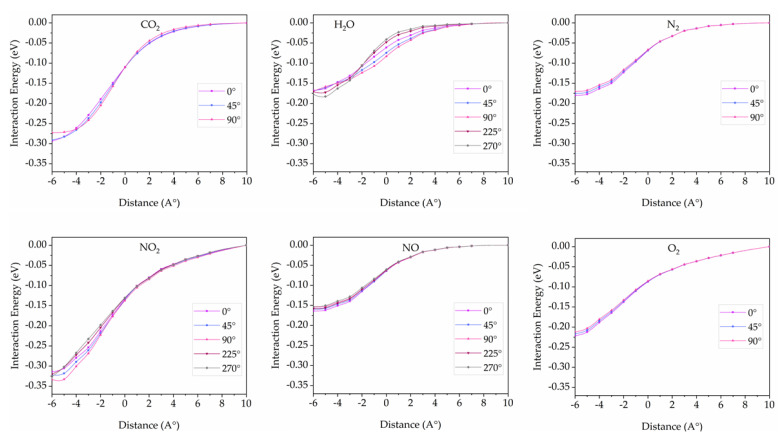
Interaction energies between 3D pillared SiC (6,6) nanostructures and gas molecules with various orientations as a function of distance.

In the case of the largest pillar diameter (6,6) (12.73 Å), no potential surface berries were found in all configurations as shown in Figure 12. All small gas molecules can easily diffuse into the 3D pillared SiC nanostructures due to their big enough hole to eliminate repulsive interactions.

## 4. Conclusions

The structural and electronic properties of new 3D pillared SiC nanostructures have been investigated by comparison with 3D pillared C nanostructures based on the SCC-DFTB method including weak interactions. The 3D pillared C nanostructures reveal high structural stability over 3D pillared SiC nanostructures. However, the average binding energies of 3D pillared SiC nanostructures are high enough for structural stability referring to a possibility in experimental growth of 3D SiC nanostructures. The 3D pillared C nanostructures exhibit metallic behavior while the SiC nanostructures display a semiconducting nature. To demonstrate an application of the 3D pillared SiC nanostructures, small gas molecules including H_2_O, CO_2_, N_2_, NO, O_2_, and NO_2_ have been intercalated into the 3D pillared SiC nanostructures with various pillar diameters. The promising results show a critical pillar diameter (5,5) (10.59 Å) that denies the diffusion of CO_2_ from the 0–50 degree configuration into 3D pillared SiC nanostructures based on the cut-off strong repulsive interaction energy of 0.2 eV. In summary, we conclude that the 3D pillared SiC nanostructures with the critical pillar diameter (5,5) (10.59 Å) have a high potential for filtering small gas molecules including H_2_O, N_2_, NO, O_2_, and NO_2_ from many bigger VOCs/gases including acetone, toluene, xylene, dimethylformamide, triethylamine, benzene, isopropanol, methanol, formaldehyde, ethanol, etc., in future applications, i.e., allowing water to pass through the 3D pillared SiC nanostructures with filter out of toluene in factories.

## Figures and Tables

**Table 1:** Summary of the pillar lengths of 3D SiC and C nanostructures before and after geometry optimization by the SCC-DFTB method.

Unit Cell	The Pillar Length (Å)			
	3D Pillared SiC Nanostructures		3D Pillared C Nanostructures	
	Before Geometry Optimization	After Geometry Optimization	Before Geometry Optimization	After Geometry Optimization
1	3.32	3.84	2.46	9.02
2	12.65	12.00	5.24	9.35
3	9.80	15.66	7.64	13.06
4	13.11	18.42	10.23	14.87
5	16.40	22.06	12.41	16.67
6	19.69	24.05	14.98	20.45
7	23.02	27.10	17.33	21.79
8	26.32	31.34	19.89	24.06

**Table 2:** Summary of the pillar diameters of 3D SiC and C nanostructures before and after geometry optimization by the SCC-DFTB method.

Armchair Nanotubes	The Pillar Diameter (Å)			
	3D Pillared SiC Nanostructures		3D Pillared C Nanostructure	
	Before Geometry Optimization	After Geometry Optimization	Before Geometry Optimization	After Geometry Optimization
(3,3)	4.01	6.25	-	-
(4,4)	5.42	8.80	-	-
(5,5)	9.11	10.59	-	-
(6,6)	10.74	12.73	8.14	9.92

**Table 3:** Summary of the diameters of gas molecules after geometry optimization by the SCC-DFTB method.

Gas Molecules	H_2_O	CO_2_	N_2_	NO	O_2_	NO_2_
Diameter (Å)	1.56	2.34	1.12	1.16	1.22	2.19

## Data Availability

Not applicable.

## Supplementary Materials

The following supporting information can be downloaded at: https://www.mdpi.com/article/10.3390/nano12111869/s1, Figure S1: The MBL analysis of the 3D pillared SiC nanostructures for the (5,5) and (6,6) pillar diameters with 6 UC pillar length; Figure S2: Interaction energies between 3D pillared SiC (6,6) nanostructures and H_2_O gas molecule with various pillar lengths and orientations; Figure S3: Interaction energies between 3D pillared SiC (3,3) nanostructures and gas molecules with various orientations as a function of distance; Figure S4: Interaction energies between 3D pillared SiC (4,4) nanostructures and gas molecules with various orientations as a function of distance; Figure S5: (a) Interaction energies between 3D pillared SiC (5,5) nanostructure and VOCs and (b) Orientation of VOCs into 3D pillared SiC (5,5) nanostructure (not to scale); Appendix SA: Command code for running fully optimization of a 3D pillared SiC nanostructure; Appendix SB: Command code for running interaction of 3D pillared SiC nanostructure and gas molecules.

## References

[1] Casady J.B., Johnson R.W. (1996). Status of silicon carbide (SiC) as a wide-bandgap semiconductor for high-temperature applications: A review. Solid-State Electron..

[2] Maboudian R., Carraro C., Senesky D.G., Roper C.S. (2013). Advances in silicon carbide science and technology at the micro- and nanoscales. J. Vac. Sci. Technol. A.

[3] Chen X., Guo X., Wu W., Fan J. (2018). Quasi-White Light-Emitting Devices Based on SiC Quantum Dots. Phys. Status Solidi (RRL)—Rapid Res. Lett..

[4] Cao Y., Dong H., Pu S., Zhang X. (2018). Photoluminescent two-dimensional SiC quantum dots for cellular imaging and transport. Nano Res..

[5] Yildirim M.A., Teker K. (2021). Self-powered fine-pattern flexible SiC single nanowire ultraviolet photodetector. J. Alloys Compd..

[6] Zekentes K., Choi J., Stambouli V., Bano E., Karker O., Rogdakis K. (2022). Progress in SiC nanowire field-effect-transistors for integrated circuits and sensing applications. Microelectron. Eng..

[7] Sun L., Wang B., Wang Y. (2018). A Novel Silicon Carbide Nanosheet for High-Performance Humidity Sensor. Adv. Mater. Interfaces.

[8] Dadkhah M., Tulliani J.-M. (2022). Nanostructured Metal Oxide Semiconductors towards Greenhouse Gas Detection. Chemosensors.

[9] Valluri S., Claremboux V., Kawatra S. (2022). Opportunities and challenges in CO_2_ utilization. J. Environ. Sci..

[10] Seesaard T., Goel N., Kumar M., Wongchoosuk C. (2022). Advances in gas sensors and electronic nose technologies for agricultural cycle applications. Comput. Electron. Agric..

[11] Rezk M.Y., Sharma J., Gartia M.R. (2020). Nanomaterial-Based CO_2_ Sensors. Nanomaterials.

[12] Usman M., Humayun M., Garba M.D., Ullah L., Zeb Z., Helal A., Suliman M.H., Alfaifi B.Y., Iqbal N., Abdinejad M. (2021). Electrochemical Reduction of CO_2_: A Review of Cobalt Based Catalysts for Carbon Dioxide Conversion to Fuels. Nanomaterials.

[13] Jacobson T.A., Kler J.S., Hernke M.T., Braun R.K., Meyer K.C., Funk W.E. (2019). Direct human health risks of increased atmospheric carbon dioxide. Nat. Sustain..

[14] García A.C., Moral-Vico J., Abo Markeb A., Sánchez A. (2022). Conversion of Carbon Dioxide into Methanol Using Cu-Zn Nanostructured Materials as Catalysts. Nanomaterials.

[15] Guzmán H., Roldán D., Sacco A., Castellino M., Fontana M., Russo N., Hernández S. (2021). CuZnAl-Oxide Nanopyramidal Mesoporous Materials for the Electrocatalytic CO_2_ Reduction to Syngas: Tuning of H_2_/CO Ratio. Nanomaterials.

[16] Pan H., Heagy M.D. (2020). Photons to Formate: A Review on Photocatalytic Reduction of CO_2_ to Formic Acid. Nanomaterials.

[17] Hiragond C.B., Powar N.S., In S.-I. (2020). Recent Developments in Lead and Lead-Free Halide Perovskite Nanostructures towards Photocatalytic CO_2_ Reduction. Nanomaterials.

[18] Desport L., Selosse S. (2022). An overview of CO_2_ capture and utilization in energy models. Resour. Conserv. Recycl..

[19] Sodeifian G., Sajadian S.A., Derakhsheshpour R. (2022). CO_2_ utilization as a supercritical solvent and supercritical antisolvent in production of sertraline hydrochloride nanoparticles. J. CO_2_ Util..

[20] Challiwala M.S., Choudhury H.A., Wang D., El-Halwagi M.M., Weitz E., Elbashir N.O. (2021). A novel CO_2_ utilization technology for the synergistic co-production of multi-walled carbon nanotubes and syngas. Sci. Rep..

[21] Ghavam S., Vahdati M., Wilson I.A.G., Styring P. (2021). Sustainable Ammonia Production Processes. Front. Energy Res..

[22] Wei Q., Lucero J.M., Crawford J.M., Way J.D., Wolden C.A., Carreon M.A. (2021). Ammonia separation from N_2_ and H_2_ over LTA zeolitic imidazolate framework membranes. J. Membr. Sci..

[23] Esser T., Wolf T., Schubert T., Benra J., Forero S., Maistros G., Barbe S., Theodorakopoulos G.V., Karousos D.S., Sapalidis A.A. (2021). CO_2_/CH_4_ and He/N_2_ Separation Properties and Water Permeability Valuation of Mixed Matrix MWCNTs-Based Cellulose Acetate Flat Sheet Membranes: A Study of the Optimization of the Filler Material Dispersion Method. Nanomaterials.

[24] Han Y., Ho W.S.W. (2021). Polymeric membranes for CO_2_ separation and capture. J. Membr. Sci..

[25] Khan F.I., Ghoshal A.K. (2000). Removal of Volatile Organic Compounds from polluted air. J. Loss Prev. Process Ind..

[26] Sivaranjanee R., Kumar P.S. (2021). A review on cleaner approach for effective separation of toxic pollutants from wastewater using carbon Sphere’s as adsorbent: Preparation, activation and applications. J. Clean. Prod..

[27] Rathi B.S., Kumar P.S. (2021). A review on sources, identification and treatment strategies for the removal of toxic Arsenic from water system. J. Hazard. Mater..

[28] Wang H., Wang M., Liang X., Yuan J., Yang H., Wang S., Ren Y., Wu H., Pan F., Jiang Z. (2021). Organic molecular sieve membranes for chemical separations. Chem. Soc. Rev..

[29] Azamat J., Khataee A. (2018). Separation of CH_4_/C_2_H_6_ Mixture Using Functionalized Nanoporous Silicon Carbide Nanosheet. Energy Fuels.

[30] Eray E., Candelario V.M., Boffa V., Safafar H., Stedgaard-Munck D.N., Zahrtmann N., Kadrispahic H., Jørgensen M.K. (2021). A roadmap for the development and applications of silicon carbide membranes for liquid filtration: Recent advancements, challenges, and perspectives. Chem. Eng. J..

[31] Elstner M., Porezag D., Jungnickel G., Elsner J., Haugk M., Frauenheim T., Suhai S., Seifert G. (1998). Self-consistent-charge density-functional tight-binding method for simulations of complex materials properties. Phys. Rev. B.

[32] Frauenheim T., Seifert G., Elsterner M., Hajnal Z., Jungnickel G., Porezag D., Suhai S., Scholz R. (2000). A Self-Consistent Charge Density-Functional Based Tight-Binding Method for Predictive Materials Simulations in Physics, Chemistry and Biology. Phys. Status Solidi (B).

[33] Gaus M., Cui Q., Elstner M. (2011). DFTB3: Extension of the Self-Consistent-Charge Density-Functional Tight-Binding Method (SCC-DFTB). J. Chem. Theory Comput..

[34] Wongchoosuk C., Wang Y., Kerdcharoen T., Irle S. (2014). Nonequilibrium quantum chemical molecular dynamics simulations of C_60_ to SiC heterofullerene conversion. Carbon.

[35] Kawamura Y., Ohta Y. (2022). Annihilation dynamics of a dislocation pair in graphene: Density-functional tight-binding molecular dynamics simulations and first principles study. Comput. Mater. Sci..

[36] Marutaphan A., Seekaew Y., Wongchoosuk C. (2017). Self-Consistent Charge Density Functional Tight-Binding Study of Poly(3,4-ethylenedioxythiophene): Poly(styrenesulfonate) Ammonia Gas Sensor. Nanoscale Res. Lett..

[37] Kondee S., Arayawut O., Pon-On W., Wongchoosuk C. (2022). Nitrogen-doped carbon oxide quantum dots for flexible humidity sensor: Experimental and SCC-DFTB study. Vacuum.

[38] Arunragsa S., Seekaew Y., Pon-On W., Wongchoosuk C. (2020). Hydroxyl edge-functionalized graphene quantum dots for gas-sensing applications. Diam. Relat. Mater..

[39] Timsorn K., Wongchoosuk C. (2019). Inkjet printing of room-temperature gas sensors for identification of formalin contamination in squids. J. Mater. Sci. Mater. Electron..

[40] Rauls E., Elsner J., Gutierrez R., Frauenheim T. (1999). Stoichiometric and non-stoichiometric (1010) and (1120) surfaces in 2H–SiC: A theoretical study. Solid State Commun..

[41] Köhler C., Hajnal Z., Deák P., Frauenheim T., Suhai S. (2001). Theoretical investigation of carbon defects and diffusion in alpha-quartz. Phys. Rev. B.

[42] Bekaroglu E., Topsakal M., Cahangirov S., Ciraci S. (2010). First-principles study of defects and adatoms in silicon carbide honeycomb structures. Phys. Rev. B.

[43] Budyka M.F., Zyubina T.S., Ryabenko A.G., Lin S.H., Mebel A.M. (2005). Bond lengths and diameters of armchair single wall carbon nanotubes. Chem. Phys. Lett..

[44] Shayeganfar F., Shahsavari R. (2016). Electronic and pseudomagnetic properties of hybrid carbon/boron-nitride nanomaterials via ab-initio calculations and elasticity theory. Carbon.

[45] Sakhavand N., Shahsavari R. (2014). Synergistic Behavior of Tubes, Junctions, and Sheets Imparts Mechano-Mutable Functionality in 3D Porous Boron Nitride Nanostructures. J. Phys. Chem. C.

[46] Humphrey W., Dalke A., Schulten K. (1996). VMD: Visual molecular dynamics. J. Mol. Graph..

[47] Weinert M., Davenport J.W. (1992). Fractional occupations and density-functional energies and forces. Phys. Rev. B.

[48] Hourahine B., Aradi B., Blum V., Bonafé F., Buccheri A., Camacho C., Cevallos C., Deshaye M.Y., Dumitrică T., Dominguez A. (2020). DFTB+, a software package for efficient approximate density functional theory based atomistic simulations. J. Chem. Phys..

[49] Wu L., Han Y., Li W., Chen S., Zhao Q., Shen L. (2022). Stable nanotube construction conditions and electronic properties of possible Si double-walled nanotubes (n_in_,m_in_)@(6,m_out_) (n_in_ =3, 4) by SCC-DFTB calculations. Mater. Chem. Phys..

[50] Ashino M., Wiesendanger R. (2017). Attractive force-driven superhardening of graphene membranes as a pin-point breaking of continuum mechanics. Sci. Rep..

[51] Seekaew Y., Arayawut O., Timsorn K., Wongchoosuk C., Yaragalla S., Mishra R., Thomas S., Kalarikkal N., Maria H.J. (2019). Chapter Nine—Synthesis, Characterization, and Applications of Graphene and Derivatives. Carbon-Based Nanofillers and Their Rubber Nanocomposites.

[52] Chabi S., Kadel K. (2020). Two-Dimensional Silicon Carbide: Emerging Direct Band Gap Semiconductor. Nanomaterials.

[53] Melchor S. (2007). TubeAnalyzer.

[54] Martín-Martínez F.J., Melchor S., Dobado J.A. (2011). Edge effects, electronic arrangement, and aromaticity patterns on finite-length carbon nanotubes. Phys. Chem. Chem. Phys..

[55] Seekaew Y., Wisitsoraat A., Phokharatkul D., Wongchoosuk C. (2019). Room temperature toluene gas sensor based on TiO_2_ nanoparticles decorated 3D graphene-carbon nanotube nanostructures. Sens. Actuators B Chem..

[56] Larina E.V., Chmyrev V.I., Skorikov V.M., D’yachkov P.N., Makaev D.V. (2008). Band structure of silicon carbide nanotubes. Inorg. Mater..

[57] Wu I.J., Guo G.Y. (2007). Optical properties of SiC nanotubes: An ab initio study. Phys. Rev. B.

[58] Alam K.M., Ray A.K. (2008). Hybrid density functional study of armchair SiC nanotubes. Phys. Rev. B.

[59] Sommerfeld T., Meyer H.-D., Cederbaum L.S. (2004). Potential energy surface of the CO_2_− anion. Phys. Chem. Chem. Phys..

[60] Shahtalebi A., Shukla P., Farmahini A.H., Bhatia S.K. (2015). Barriers to diffusion of CO_2_ in microporous carbon derived from silicon carbide. Carbon.

[61] Farmahini A.H., Shahtalebi A., Jobic H., Bhatia S.K. (2014). Influence of structural heterogeneity on diffusion of CH_4_ and CO_2_ in silicon carbide-derived nanoporous Carbon. J. Phys. Chem. C.

[62] Zhao J., Buldum A., Han J., Lu J.P. (2002). Gas molecule adsorption in carbon nanotubes and nanotube bundles. Nanotechnology.

[63] Králik M. (2014). Adsorption, chemisorption, and catalysis. Chem. Pap..

[64] Mehmood F., Kara A., Rahman T.S., Bohnen K.P. (2006). Energetics of CO on stepped and kinked Cu surfaces: A comparative theoretical study. Phys. Rev. B.

[65] Yildirim H., Kara A. (2013). Effect of van der Waals interactions on the adsorption of olympicene radical on Cu (111): Characteristics of weak physisorption versus strong chemisorption. J. Phys. Chem. C.

