# Supplementary material for: Structures, Electronic Properties, and Gas Permeability of 3D Pillared Silicon Carbide Nanostructures
Source: Nanomaterials (Basel). 2022 May 30;12(11):1869. doi: 10.3390/nano12111869 (PMC9182379; doi:10.3390/nano12111869)
Supplement: Supplementary file 1 [file nanomaterials-12-01869-s001.zip › nanomaterials-1739526-supplementary-done.pdf]

nanomaterials

Supporting Information

Structures, Electronic Properties, and Gas Permeability of 3D Pillared Silicon Carbide Nanostructures

Onsuda Arayawut [1], Teerakiat Kerdcharoen [2] and Chatchawal Wongchoosuk [1,*]

[1] Department of Physics, Faculty of Science, Kasetsart University, Chatuchak, Bangkok 10900, Thailand; onsuda.ar@gmail.com
[2] Department of Physics, Faculty of Science, Mahidol University, Bangkok 10400, Thailand; teerakiat.ker@mahidol.ac.th
* Correspondence: chatchawal.w@ku.ac.th

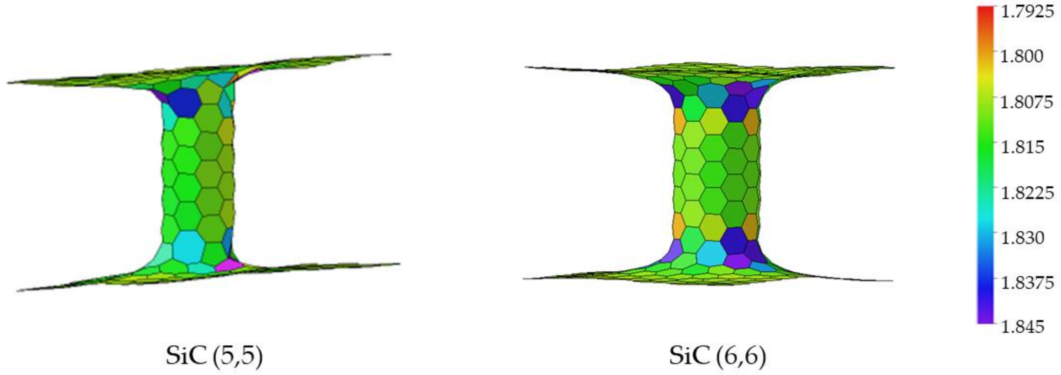

Figure S1. The MBL analysis of the 3D pillared SiC nanostructures for the pillared (5,5) and (6,6) diameter with 6 UC pillared length.

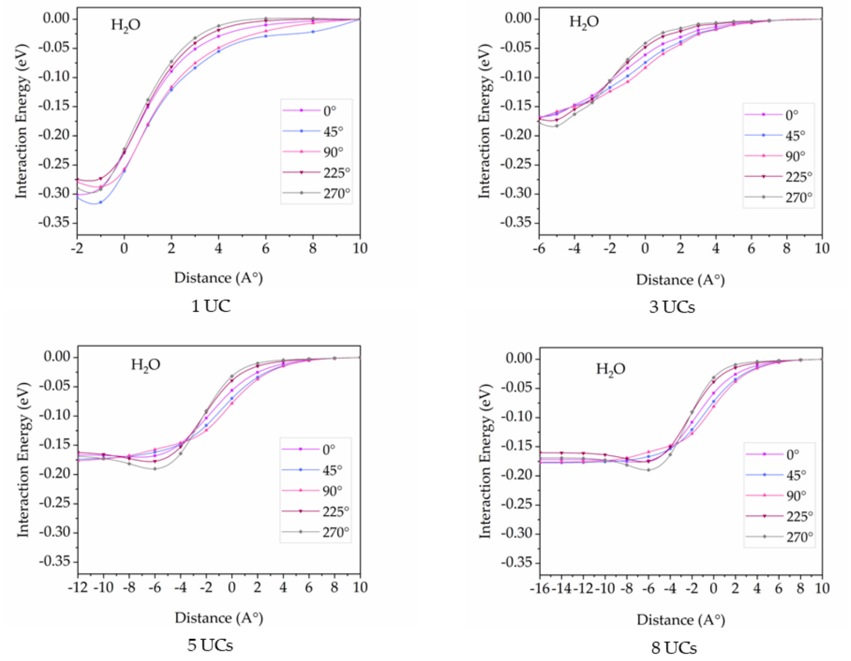

Figure S2. Interaction energies between 3D pillared SiC (6,6) nanostructures and H_2O gas molecule with various pillared lengths and orientations.

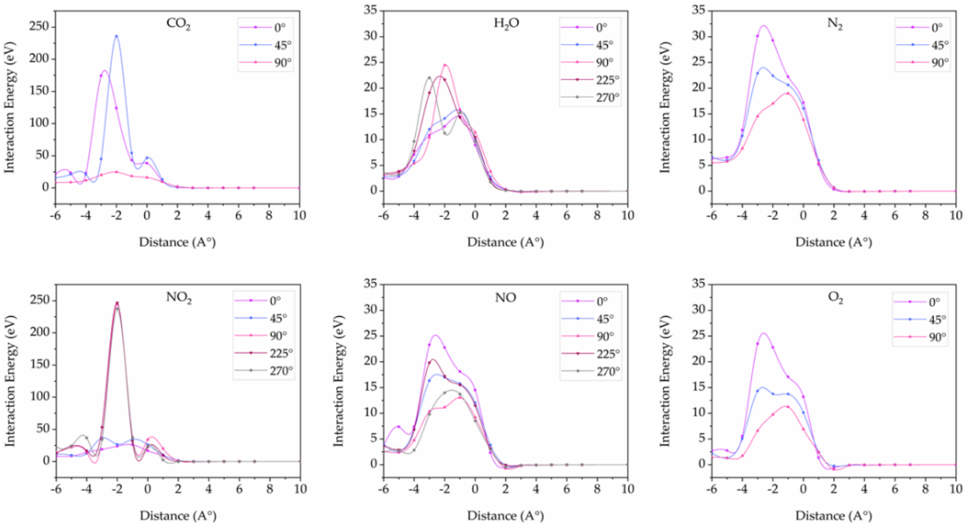

Figure S3. Interaction energies between 3D pillared SiC (3,3) nanostructures and gas molecules with various orientations as a function of distance.

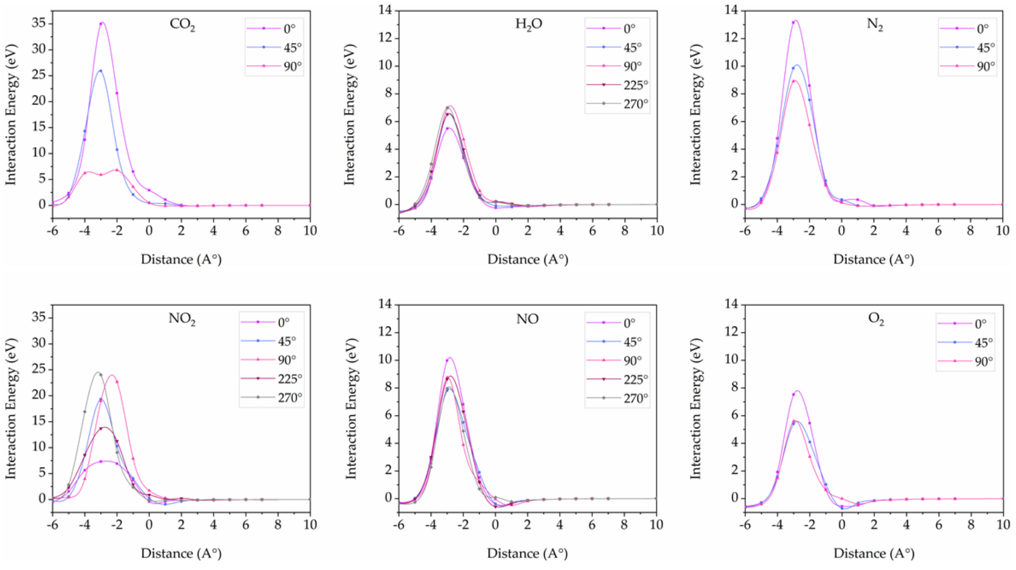

Figure S4. Interaction energies between 3D pillared SiC (4,4) nanostructures and gas molecules with various orientations as a function of distance.

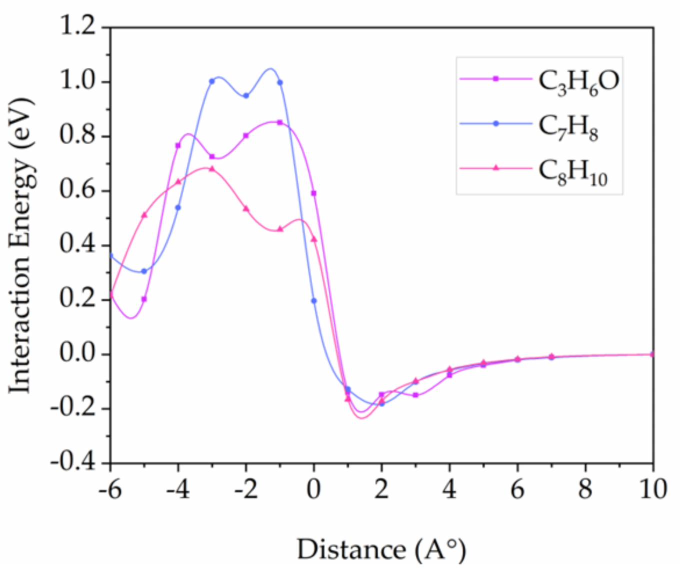

(a)

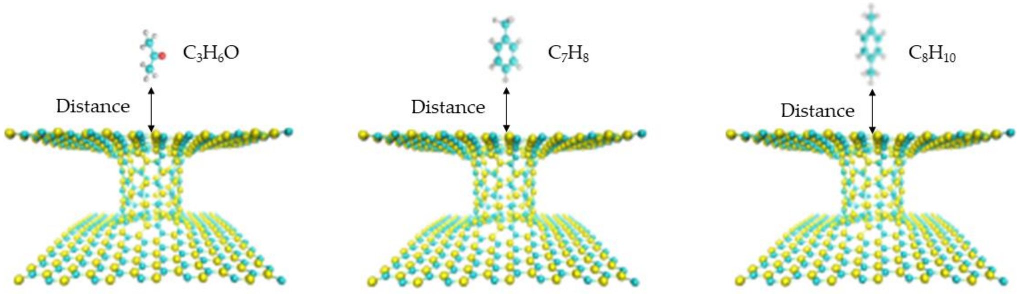

(b)

Figure S5. (a) Interaction energies between 3D pillared SiC (5,5) nanostructure and VOCs and **(b)** Orientation of VOCs into 3D pillared SiC (5,5) nanostructure (Not to scale).

Appendix SA Command code for running fully optimization of a 3D pillared SiC nanostructure

```
Geometry = GenFormat {
<<< "1.gen"
}
Hamiltonian = DFTB {
  SCC = Yes
  SCCTolerance = 1e-6
  MaxSCCIterations = 1000
  Mixer = Broyden {}
  MaxAngularMomentum = {
    C = "p"
    Si = "p"
  }
  Charge = 0.0
  SpinPolarisation ={}
  Filling = Fermi {
    Temperature [K] = 300
  }
Dispersion = LennardJones {
```

```
Parameters {
 Si {
Distance [AA] = 4.295
Energy [kcal/mol] = 0.402
}
C {
Distance [AA] = 3.851
Energy [kcal/mol] = 0.105
}
}
}
 SlaterKosterFiles = {
    C-C = "/opt/programs/DFTB+/TB-paras/pbc-0-3/C-C.skf"
    C-Si = "/opt/programs/DFTB+/TB-paras/pbc-0-3/C-Si.skf"
    Si-C = "/opt/programs/DFTB+/TB-paras/pbc-0-3/Si-C.skf"
  Si-Si = "/opt/programs/DFTB+/TB-paras/pbc-0-3/Si-Si.skf"
 }
KPointsAndWeights = SupercellFolding {
1   0 0
0   1 0
0   0   1
0.0 0.0 0.0
}
  }
Driver = ConjugateGradient {
  LatticeOpt = Yes
  MovedAtoms = 1:-1
  MaxForceComponent = 1.0e-4
  MaxSteps = 10000
  OutputPrefix = "geo_end"
  Constraints = {}
  AppendGeometries = Yes
}
```

Appendix SB Command code for running interaction of 3D pillared SiC nanostructure and gas molecule

```
Geometry = GenFormat {
<<< "1.gen"
}
Hamiltonian = DFTB {
  SCC = Yes
  SCCTolerance = 1e-6
  MaxSCCIterations = 1000
  Mixer = Broyden {}
  MaxAngularMomentum = {
    C = "p"
   Si = "p"
    O = "p"
  }
  Charge = 0.0
  SpinPolarisation ={}
  Filling = Fermi {
    Temperature [K] = 300
  }
```

```
Dispersion = LennardJones {
 Parameters {
 C {
 Distance [AA] = 3.851
 Energy [kcal/mol] = 0.105
 }
 Si {
 Distance [AA] = 4.295
 Energy [kcal/mol] = 0.402
 }
 O {
 Distance [AA] = 3.500
 Energy [kcal/mol] = 0.060
 }
 }
 }
  SlaterKosterFiles = {
     C-C = "/opt/programs/DFTB+/TB-paras/pbc-0-3/C-C.skf"
     Si-Si = "/opt/programs/DFTB+/TB-paras/pbc-0-3/Si-Si.skf"
     O-O = "/opt/programs/DFTB+/TB-paras/pbc-0-3/O-O.skf"
     C-Si = "/opt/programs/DFTB+/TB-paras/pbc-0-3/C-Si.skf"
     Si-C = "/opt/programs/DFTB+/TB-paras/pbc-0-3/Si-C.skf"
     C-O = "/opt/programs/DFTB+/TB-paras/pbc-0-3/C-O.skf"
     O-C = "/opt/programs/DFTB+/TB-paras/pbc-0-3/O-C.skf"
     Si-O = "/opt/programs/DFTB+/TB-paras/pbc-0-3/Si-O.skf"
     O-Si = "/opt/programs/DFTB+/TB-paras/pbc-0-3/O-Si.skf"
  }
KPointsAndWeights = SupercellFolding {
1   0 0
0   1 0
0   0   1
0.0 0.0 0.0
}
  }
Driver = {}
```
